# Gibberellin A_1_ Metabolism Contributes to the Control of Photoperiod-Mediated Tuberization in Potato

**DOI:** 10.1371/journal.pone.0024458

**Published:** 2011-09-22

**Authors:** Jordi Bou-Torrent, Jaime F. Martínez-García, José Luis García-Martínez, Salomé Prat

**Affiliations:** 1 Centre de Recerca en Agrigenòmica (CRAG) CSIC-IRTA-UAB, Bellaterra, Spain; 2 Institució Catalana de Recerca i Estudis Avançats, Barcelona, Spain; 3 Instituto de Biologia Molecular y Celular de Plantas, UPV-CSIC, Valencia, Spain; 4 Centro Nacional de Biotecología-CSIC, Madrid, Spain; Instituto de Biología Molecular y Celular de Plantas, Spain

## Abstract

Some potato species require a short-day (SD) photoperiod for tuberization, a process that is negatively affected by gibberellins (GAs). Here we report the isolation of *StGA3ox2*, a gene encoding a GA 3-oxidase, whose expression is increased in the aerial parts and is repressed in the stolons after transfer of photoperiod-dependent potato plants to SD conditions. Over-expression of *StGA3ox2* under control of constitutive or leaf-specific promoters results in taller plants which, in contrast to *StGA20ox1* over-expressers previously reported, tuberize earlier under SD conditions than the controls. By contrast, *StGA3ox2* tuber-specific over-expression results in non-elongated plants with slightly delayed tuber induction. Together, our experiments support that *StGA3ox2* expression and gibberellin metabolism significantly contribute to the tuberization time in strictly photoperiod-dependent potato plants.

## Introduction

Potato tubers differentiate at the tip of stolons (underground stems), which upon tuber induction stop growing longitudinally and start swelling. They serve as vegetative propagation organs, as well as energy reservoir for the future new plant. Potato tubers are a rich source of carbohydrates widely used in food and industry, being the third crop in economical importance after wheat and rice (faostat.fao.org). Hence, it is of great agronomic interest to unveil signals that control tuber formation.

The control of tuber formation is complex and environmental factors such as photoperiod, temperature, and shortage in nitrogen supply are known to have an important effect [1]. Short days (SD, 8 h light/16 h dark) favor tuberization whereas long days (LD, 16 h light/8 h dark) delay this developmental process. Sensitivity to day length depends on the genetic background: whereas most commercial cultivars of *Solanum tuberosum* were bred to be relatively independent on day length for tuberization, wild potato species like *S. demissum* and several varieties of *S. tuberosum* ssp. *andigena* are strictly dependent on SD for tuber formation [2]. These wild species tuberize under SD conditions, but do not form tubers when grown under LDs.

Current evidence supports the existence of at least a photoperiod- and a GA-dependent pathway in controlling potato tuberization [3], [4], [5]. Somehow these independent pathways interact and the balance between their positive and negative effects determines tuberization. Therefore, some cross-talk between both pathways is occurring.

**Figure 1:**
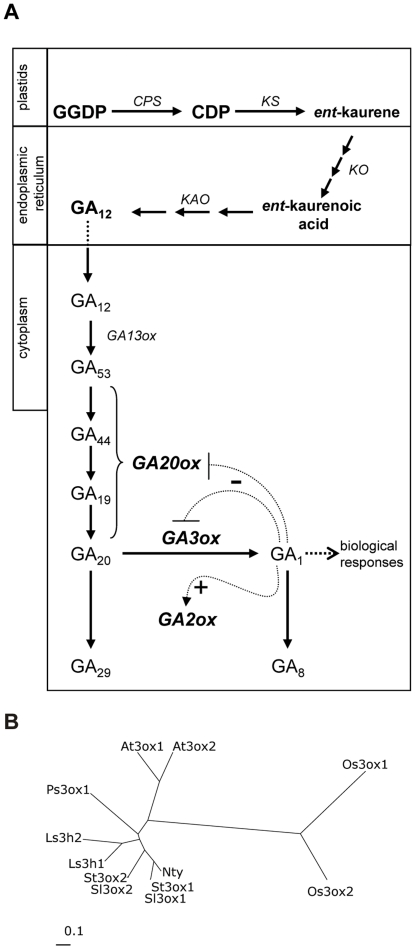
Major reactions in GA biosynthetic pathway and phylogenetic analysis of GA 3-oxidases. (**A**) The pathway is classified into three groups based on the enzymatic activity and subcellular localization. Plastidial terpene cyclases convert the precursor geranylgeranyl diphosphate (GGDP) into *ent*-copalyl diphosphate (CDP), and subsequently into *ent*-kaurene, in a two-step process catalyzed by *ent*-copalyl diphosphate synthase (*CPS*) and *ent*-kaurene synthase (*KS*). Cytochrome P-450 mono-oxygenases such as *ent*-kaurene oxidase (*KO*), *ent*-kaurenoic acid oxidase (*KAO*) yield GA_12_. Cytoplasmic dioxygenases are responsible for the latest steps and conversion to bioactive gibberellins and their inactive catabolites. GA_12_ in potato is early 13-hydroxylated by GA 13-oxidase (*GA13ox*). As indicated by curved dotted T-lines or arrows emerging from the bioactive GA_1_, *GA20ox* and *GA3ox* corresponding transcripts are under negative feed-back regulation, whereas *GA2ox* mRNA is under positive feed-forward regulation by GA_1_. (**B**) Neighbor-joining tree obtained with the alignment of the amino acid sequences: St3ox1 and St3ox2 correspond to potato *StGA3ox1* (FJ792644) and *StGA3ox2* (FJ792643); At3ox1 and At3ox2 to arabidopsis *AtGA3ox1* (NM101424) and *AtGA3ox*2 (NM106683); Ps3ox1 to pea *PsGA3ox1* (AF010167), Ls3h1 and *Ls3h*2 to lettuce *LsGA3ox1* (AB012205) and *LsGA3ox2* (AB012206); *Le3ox1* and *Le3ox*2 to tomato *SlGA3ox1* (AB010991) and *SlGA3ox2* (AB010992), tobacco *Nt*y is *NtGA3ox1* (AB032198), and *Os3ox1* and *Os3ox2* are rice genes *OsGA3ox1* and *OsGA3ox2* (AB054084 and AB056519).

Gibberellins (GAs) have also been shown to regulate tuberization. For instance, greater GA content is observed in stolons of plants grown under non-inductive LDs, whereas a decrease in GA activity is found when leaves are exposed to inductive SD [6], [7]. Increased GA activity has also been observed in response to high temperatures or continuous nitrate supply, conditions that prevent tuber formation [8], [9]. The dwarf *ga1* mutant of the photoperiod-dependent *S. tuberosum* ssp *andigena*, which appears to be blocked in the GA biosynthesis 13-hydroxylation step (Figure 1A), forms tubers after culture for several months under LD conditions [10]. Treatment of potato ssp. *andigena* plants with GA synthesis inhibitors induces tuberization in LD [11]. Altogether, the content of GAs is greater in conditions that inhibit tuberization and conversely a reduction in GA content might induce tuberization even under non-inductive conditions, which led to the generally accepted conclusion that GAs inhibit tuberization.

GA 20-oxidases and GA 3-oxidases catalyze the last two steps of active GA biosynthesis, and GA 2-oxidase their conversion to inactive catabolites (Figure 1A). These three types of enzymes are encoded by small families of *GA20ox*, *GA3ox* and *GA2ox* genes [12], [13]. All three enzymatic steps are thought to be main sites of GA biosynthesis regulation (Figure 1A) [14]. *GA20ox* and *GA3ox* expression is under feed-back regulation by GA_1_/GA_3_, whereas *GA2ox* expression is under feed-forward regulation by bioactive GAs [12]. In addition, *GA20ox* expression is also regulated in response to day length in several plant species [12], [15], [16], [17]. In Arabidopsis and spinach, for example, bolting in response to LD conditions is associated to greater levels of *GA20ox* expression [18], [19]. In potato, by contrast, levels of the three *StGA20ox* transcripts were not greater in the leaves of potato plants grown under non-inductive (SD+NB or LD) conditions compared to plants grown under tuber-inducing (SD) conditions. Therefore, it was concluded that changes in GA levels observed during tuber induction do not result from regulated expression of this biosynthetic activity [20].

Light regulation of GA biosynthetic gene expression or activities other than GA 20-oxidases has also been reported [15]. For instance, enhanced synthesis of *ent*-kaurene under long photoperiods has been observed in the LD plants spinach (*Spinacia oleracea*) and *Agrostemma githago* [21]. In Arabidopsis and lettuce (*Lactuca sativa*) seeds, expression of the GA 3-oxidases AtGA3ox2 (*GA4H*) and *Ls3ox1* genes is induced by seed exposure to red light and this effect is reversed by far-red light treatment. Hence, red light appears to promote GA_1_ synthesis in these seeds by inducing GA 3-oxidase expression via phytochrome action [22], [23]. Transfer of etiolated pea (*Pisum sativum*) seedlings to light, in its turn, was found to down-regulate expression of *PsGA3ox1* (Mendel's *Le* gene) and up-regulate *PsGA2ox2*, encoding for a deactivating GA 2-oxidase [24], [25]. In cowpea (*Vigna sinensis*), end-of-day far-red treatments, that stimulate epicotyl elongation, resulted in an increase in GA_1_ levels by inhibiting at least GA 2-oxidase activity in the epicotyl tissues [26]. In potato, *StGA2ox1* expression is strongly up-regulated during the early stages of potato tuber development, prior to visible swelling. Characterization of transgenic potato plants with altered levels of *StGA2ox1* led to propose a role for this gene in early tuber initiation by reducing GA levels in the subapical stolon region at the onset of tuberization, thereby facilitating normal tuber development and growth [27]. Therefore, the regulated expression of a potato biosynthetic activity different from GA 20-oxidase may modulate reduced GA synthesis under SD conditions and/or contribute to tuberization.

To address the aforementioned possibility, we set out to clone additional GA biosynthetic genes from *S. tuberosum* ssp. *andigen*a. In this manuscript, we report the functional characterization of *StGA3ox*2, a gene encoding a GA 3-oxidase from potato, whose expression is differentially regulated in aerial parts and stolons after transfer to SD inductive conditions. Our experiments suggest the importance of this activity in the control of photoperiod-induced potato tuberization.

## Materials and Methods

### Plant material and growth conditions

Photoperiodic *Solanum tuberosum* ssp. *andigena* plants were propagated *in vitro* in MS media supplemented with 20 g·L^−1^ sucrose before being transferred to soil, or from tubers. Plant height analysis was performed with plants at the 14-leaf stage, grown in the greenhouse under LD conditions (16 h light/8 h darkness, 22°C). Internode length was measured as reported before [28]. Transformation of potato plants was performed as described elsewhere [29]. About 40 independent transgenic lines were regenerated for each construct, transferred to soil and analyzed at the RNA level. For tuber induction measurements, plants were grown in the greenhouse under LD conditions until they reached a 14-leaf stage and then were transferred to growth chambers under SD conditions (8 h light/16 h dark, 22°C, SD inducing conditions) or to SD with a light treatment of 30 min or night-break in the middle of the dark period (SD+NB, non-inducing conditions). Light intensity in the growth chambers was about 200 µmol·m^−2^·s^−1^ provided by high-pressure sodium lamps SON-T AGRO 400 (Philips).

### Potato *GA3ox* PCR amplification and plasmid constructs

Since potato and tomato cDNA collections were very limited at the time this work was initiated, in order to amplify potato cDNAs encoding GA 3-oxidases, degenerated oligonucleotides were designed upon conserved sequences of the *AtGA3ox1 (GA4)* and *PsGA3ox2* (*Le)* genes from Arabidopsis and pea [30], [31], [32] and used in PCR. Primers H5 (5′-TGG-GGI-(AG)(CT)I-TT(CT)-CA(AG)-(AG)T-3′) and H7 (5′-GT(AG)-TGI-G(GC)I-GCI-A(AG)I-CCC-AT-3′), complementary to the regions 78-WGAFQI-83 and 227-MGLAAHT-233 in *AtGA3ox1* respectively yielded a band of expected size. PCR fragments were cloned in pGEM T-easy (Promega) and sequenced. A first strand cDNA obtained from mRNA extracted from leaves of the potato GA deficient mutant *ga1* [33] was used as template for PCR amplification. The subcloned *B3ox* PCR product, which corresponded to a transcript with negative feed-back regulation (Figure S1), was used to screen a cDNA library constructed from leaves of the potato *ga1* mutant [20]. Several positives clones were isolated and sequenced (Results S1, Figure S2A), resulting in the isolation of one single full cDNA clone, closely related to the later reported tomato GA 3-oxidase *SlGA3ox2* [34]. Hence, the isolated potato clone was designated as *StGA3ox2* (gb|ACN89834). Specific primers B8 (5′-AGT-CCT-TCA-AGA-ATC-3′) and B9 (5′-GTA-AGT-GTC-ACT-AGA-GAA-T-3′), complementary to the tomato *SlGA3ox1* [34], were used to amplify a second potato GA 3-oxidase-encoding gene, which was designated as *StGA3ox1* (gb|ACN89835).

To produce StGA3ox2 protein in *E. coli* its coding sequence was amplified using oligonucleotides 3B5 (5′-AAT-TTT-CTA-CTC-ATA-TGC-CTT-CAA-3′) and 3B3 (5′-GCC-GCT-CGA-GGC-CTA-CTT-GGA-GAC-3′) bearing *Nde*I and *Xho*I restrictions sites. The PCR product was conveniently digested and ligated to the pET28 (Novagen) expression vector restricted with the same pair of enzymes. The clone pET3ox2 was isolated after PCR selection and its sequence was confirmed.

To over-express *StGA3ox2* in transgenic potato plants the isolated cDNA clone number 10 (Figure S2A) was digested with *Ec*oRI (containing the complete coding region and part of the 3′ non-coding region), filled-in and cloned into three different plant binary vectors: pBinAR [35], pBinA6 [36], and pB33 [29], directing expression under control of the constitutive CaMV*35S* promoter (lines *35S:3ox2*), the green-tissue specific promoter *StLS1* (lines *LS1:3ox2*), and the tuber-specific patatin promoter (lines *Tub1:3ox2*) respectively.

### RNA isolation and gel-blot analysis

Total RNA was extracted from different potato plant tissues according to the method described [37]. Electrophoresis, northern analysis and labeling of the probes were carried out as described [28].

### Gibberellin content analysis

GA content was determined from the apical part of potato shoots (including the shoot apex and the four youngest internodes and leaves). Tissue was frozen in liquid N_2_, kept at −80°C until use for GA extraction and quantification by HPLC and GC-MS as described elsewhere [38].

### GA 3-oxidase recombinant protein and activity assay

BL21 *E. coli* competent cells were transformed with pET3ox2. Cultures were grown at 30°C until OD_600_ = 0.4 and recombinant StGA3ox2 protein accumulation was induced with 1 mM IPTG for 2 h shaking at 30°C. Aliquots were taken before and after induction, to check the accumulation of the recombinant protein in the soluble fraction. Cell lysates producing StGA3ox2 were prepared by resuspending initial 50 mL pelleted cultures in 2 mL TrisHCl pH = 7.5, 4 mM DTT and 1 mg/mL lysozime. After 15 min incubation at room temperature and sonication, extracts were ultracentrifuged at 35.000 rpm for 30 min at 4°C. Supernatants were frozen for activity assays. 85 µL aliquots of these cell lysates were incubated at 30°C for 2 h with either [^3^H]-GA_20_ and [^2^D]-GA_20_ or with [^14^C]-GA_9_ in a total volume of 100 µL containing 4 mM 2-oxoglutarate, 4 mM ascorbate, 0.5 mM ferrous sulphate, 4 mM DTT and 2 mg/mL BSA, as described in [39]. The reaction mixture was analyzed by HPLC, detecting the radioactivity of each sample in a Beckman scintillation counter. Fractions at elution times corresponding to GA_1_ and GA_4_ were analyzed by GC-MS for confirmation of the reaction products.

## Results

### Potato GA 3-oxidase is encoded by at least two genes

When this work was initiated, potato and tomato cDNA collections were very limited and no information was available on GA 3-oxidase-encoding genes from these species. Therefore cDNA prepared from leaves of the *S. tuberosum* ssp *andigena ga1* mutant was used as template to amplify *StGA3ox* genes, because this mutant is supposed to accumulate high levels of the GA 3-oxidase-encoding transcripts due to negative feed-back regulation. As a result (check Methods for detailed information), we identified a full length clone with a higher percentage of homology to the pea *PsGA3ox1* (*Le)* and the Arabidopsis *AtGA3ox2* (*GA4*) genes [30], [31], [32] than to any other 2-oxoglutarate dependent dioxygenase sequence available at that moment, thus suggesting that it encoded a potato GA 3-oxidase. The sequences from tomato (*Solanum lycopersicum*), *SlGA3ox1* and *SlGA3ox2*, were later reported [34], and because the potato full length clone shared 92% identity with *SlGA3ox2*, it was designated as *StGA3ox2*. Based on the nucleotide sequence of the *SlGA3ox1* gene and using RT-PCR and specific primers (B8 and B9) on potato RNA as a template, a new cDNA was cloned, named as *StGA3ox1*, which included the complete coding region corresponding to this gene (Figure S2B). From these results we conclude that potato, like tomato, has at least two independent genes encoding GA 3-oxidase enzymes as shown in the phylogenetic tree of Figure 1B.

### *StGA3ox1* and *StGA3ox2* show a differential spatial pattern of expression

**Figure 2:**
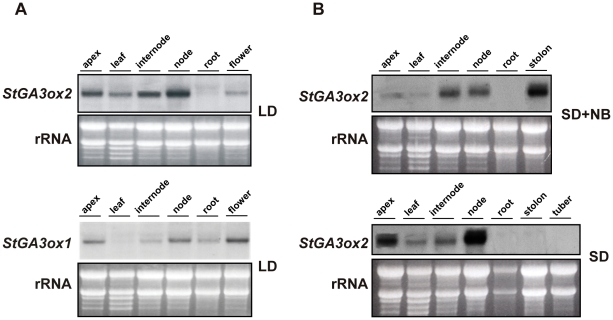
Tissue specific expression pattern of *StGA3ox1* and *StGA3ox2* transcripts. (**A**) Analysis of tissue-specific pattern of expression of clones *StGA3ox1* and *StGA3ox*2, at 14-leaf developmental stage of plants grown in the greenhouse (LD). (**B**) Expression of *StGA3ox2* in different organs from plants grown in LD until the 14-leaf stage and then entrained to SD+NB or to SD for three weeks. 30 µg of total RNA from each sample was electrophoresed and equal loading on the gel was assessed by ethidium bromide staining, representative pictures are shown out of at least two independent *Northern* analysis.

The expression of the *StGA3ox1* and *StGA3ox2* genes was analyzed by Northern blot using RNAs from different organs of *S. tuberosum* ssp *andigena* plants (leaf-14 stage) grown under non-inducing LD conditions (Figure 2A). Because at this developmental stage plants had very few or no stolons, these organs were not harvested in our analyses. Different patterns of expression were observed for the two *StGA3ox* genes. Relatively high levels of the *StGA3ox2* transcript were found in the apex, internodes and stem nodes, lower levels in flowers and leaves and almost no expression in roots. *StGA3ox*1 transcripts were detected mainly in flowers and, to a lower extent, in apex, nodes, internodes and roots. Almost no expression was detected in leaves (Figure 2A). Thus, whereas *StGA3ox2* was expressed in most vegetative tissues, *StGA3ox1* transcripts seemed to be more abundant in floral organs. Consequently, we focused on the study of *StGA3ox2*. Consistent with our results, data on the expression levels of the two *StGA3ox* genes in non-tuberizing stolon tips detected expression of only *StGA3ox2* [27].

### *StGA3ox2* encodes a functional potato GA 3-oxidase

**Figure 3:**
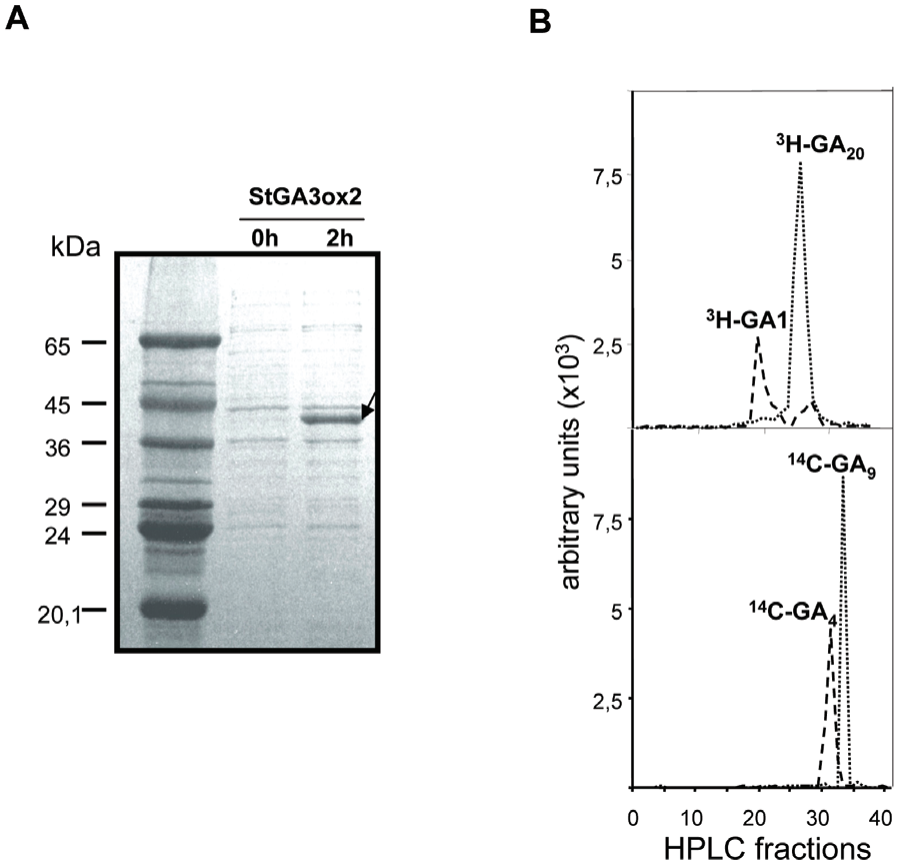
GA 3-oxidase activity assay in recombinant *E.coli* lysates expressing StGA3ox2 protein. (**A**) Recombinant StGA3ox2 accumulated in BL21 *E.coli* growing at 30°C. After 2 h induction with IPTG, we detected a band with a molecular weight of 43 kDa corresponding to StGA3ox2. (**B**) HPLC chromatogram after 2 h incubation of *E.coli* lysates expressing recombinant StGA3ox2 feeded with the radiolabeled putative substrates ^3^H-GA_20_ and ^14^C-GA_9_. The corresponding products peaked at the ^3^H-GA_1_ and ^14^C-GA_4_ corresponding elution times.

In order to assess the GA 3-oxidase activity of the StGA3ox2 protein, we obtained pET3ox2 clone, containing the coding sequence of *StGA3ox2* in frame in a pET28 (Novagen) expression vector. *E. coli* BL21 strain was transformed with the pET3ox2 clone and grown at 30°C. The recombinant protein was allowed to accumulate by induction with IPTG for 2 h (Figure 3A). Crude extracts of the expression cultures were incubated for 2 h at 30°C in the presence of the radiolabeled substrates, either [^3^H]-GA_20_ or ^14^C-GA_9_. HPLC analysis of the reaction products resulted with radioactivity peaks at the same elution time as ^3^H-GA_1_ and ^14^C-GA_4_ in each reaction (Figure 3B). These fractions were further identified by GC-MS as GA_1_ and GA_4_ (data not shown), thus confirming that *StGA3ox2* encodes a functional GA 3-oxidase.

### Expression of StGA3ox2 in the stolons is strongly repressed under tuber-inducing (SD) conditions

In previous studies no major changes were detected in the levels of expression of the *StGA20ox* genes between potato plants induced (SD) and non-induced (SD+NB or LD) to tuberize [20]. Therefore, we have investigated whether photoperiod regulated the expression of *StGA3ox2* in different organs of 14-leaf plants grown for 3 additional weeks under inductive (SD) and non-inductive (SD+NB) conditions (Figure 2B). In plants grown under non-inductive conditions (SD+NB or LD) levels of *StGA3ox2* transcript were similar but not identical (compare Figs. 2A and 2B). Remarkably high levels of *StGA3ox2* mRNA were detected in the underground stolons. By contrast, in plants grown under inductive SD conditions, *StGA3ox2* expression was clearly up-regulated in apex, leaves and nodes. The most conspicuous change in gene expression however was found in the stolons, where *StGA3ox2* expression was completely repressed, with no transcript detected either in stolons of plants induced to tuberize or tubers already formed (Figure 2B). Since exposure of potato plants to SD tuber-inducing conditions results in a large decrease in stolon GA levels [7], our correlative data led us to hypothesize that the repression of this GA 3-oxidase activity participates in the reduction of stolon GA content associated with tuber induction.

### Expression of *StGA3ox2* in the aerial part of the plant is diurnally regulated and is increased under tuber-inducing (SD) conditions

**Figure 4:**
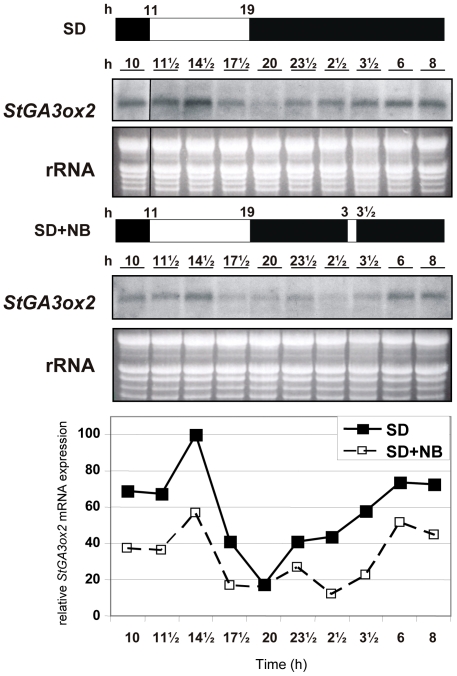
Analysis of diurnal rhythm of *StGA3ox2* expression in leaves. Plants at 14-leaf stage were entrained to SD (8 h light/16 h dark) or SD+NB conditions (8 h light/16 h dark with a 30 min light break in the middle of the night) for 3 weeks. Leaf samples were harvested at the time points indicated, approximately every 2 to 3 hours, for a period of 24 hours. The lanes come from the same gel, a dividing line (in SD panels) indicates intervening lanes have been removed. Films were quantified by densitometric scanning and levels of transcript were normalized to the sample with the strongest hybridization signal (14:30 h in SD), to which we assigned an arbitrary value of 100. 30 µg of total RNA from each sample was electrophoresed and equal loading on the gel was assessed by ethidium bromide staining, representative pictures are shown out of at least two independent *Northern* analysis.

The levels of *StGA3ox2* transcript in leaves were found to be dependent on the time of the day at which leaves were sampled. To analyze whether this was due to a diurnal rhythm of expression, we studied the accumulation of *StGA3ox2* messenger in potato leaves of plants grown under inducing (SD) and non-inducing (SD+NB) conditions, over a period of 24 h. Similar patterns of transcript abundance were observed in plants entrained to SD or SD+NB conditions within the 24 h period (Figure 4). The expression of *StGA3ox2* fluctuated during the day, with transcript levels reaching a peak around 3 h after starting the light period (lights were switched on at 11:00 h and maximal levels of transcript were detected at 14:30 h) and a valley around 2 h after lights switch-off, after which they gradually recovered during the night period (Figure 4). Although the pattern of *StGA3ox2* transcript accumulation was similar in leaves of plants entrained to SD or SD+NB conditions, levels of *StGA3ox2* mRNA were higher in SD- than SD+NB-grown plants over the entire 24 h period. This agrees with the results shown in Figure 2B and suggests that, in contrast with that observed in the underground stolons, increased levels of *StGA3ox2* mRNA occur in leaves and other aerial part of the shoot of plants entrained to SD conditions compared to plants grown under SD+NB non-inducing conditions.

### Constitutive over-expression of *StGA3ox2* in the aerial parts results in elongated plants and early tuberization under SD conditions

**Figure 5:**
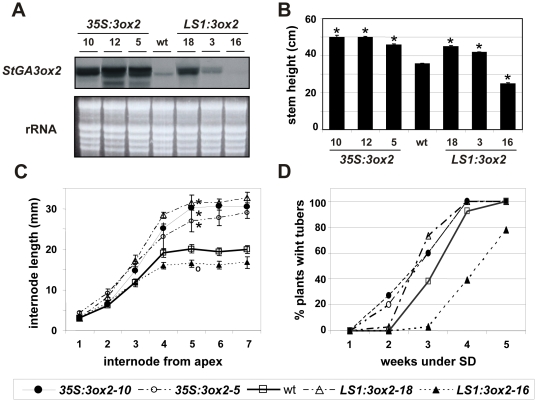
Over-expression of *StGA3ox2* under constitutive (*35S:3ox2*) and leaf-specific (*LS1:3ox2*) promoters. (**A**) RNA blot analysis from LD-grown shoots, 30 µg of total RNA from each sample was electrophoresed and equal loading on the gel was assessed by ethidium bromide staining, a representative picture is shown out of at least two independent *Northern* analysis. (**B**) Stem height and (**C**) Internode length of overexpressing lines, wt and cosupressed line grown under LD conditions. Mean value obtained from 10–15 individual plants from each line. Errors bars indicate SE, asterisks (*) highly significant differences (P<0,01) and circles ( ) significant differences (P<0,05) compared to wt and for the 5^th^ internode in C. (**D**) Tuber induction data, represent the average of 10–15 independent replicates for each line from a representative experiment out of three with similar results.

**Figure 6:**
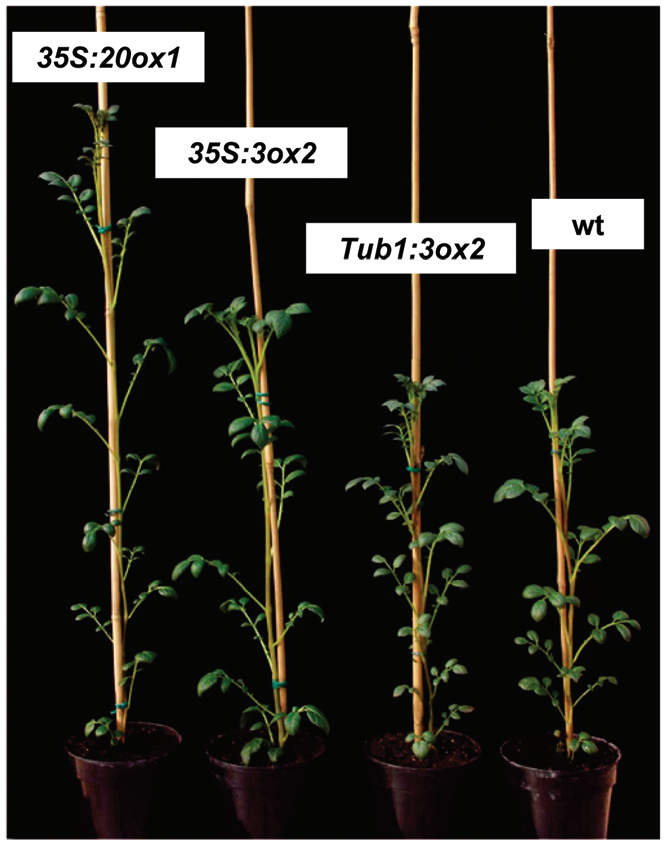
Phenotype of representative GA 3-oxidase and GA 20-oxidase transgenic plants. Plant height of the constitutive *35S:3ox2–10* (*35S:3ox2*) and the tuber-specific *Tub1:3ox2–30* (*Tub1:3ox2*) over-expressers is shown in comparison to that of lines over-expressing the GA 20-oxidase activity (*35S:20ox1*) or wild-type (wt) plants. The number of nodes was the same in all plants.

The putative regulatory role of GA biosynthesis catalyzed by *StGA3ox2* led us to investigate its function in the photoperiod-dependent tuberization. Plants over-expressing the *StGA3ox2* transcript under the control of the constitutive *35S* promoter (*35S:3ox2* lines) or the green tissue specific *StLS1* promoter (*LS1:3ox2* lines) were obtained. The *StLS1* promoter drives expression to green tissues of the leaves and stem [36]. Several transformants were regenerated that accumulated higher levels of *StGA3ox2* transcript than the controls (Figure 5A). These lines exhibited an elongated phenotype as compared to the wild-type controls (Figure 5B and 6), with taller stems caused by an increase in internode length and not by an elevated number of internodes (see Figure 5C). GA content was quantified in shoots (leaves and stems) of four different *StGA3ox2* over-expressing lines. Higher levels of GA_1_ (ranging from 1.3 to 2.2 fold-increase), and much lower levels of its 13-hydroxylated precursor GA_20_ and of GA_29_ (a GA_20_ catabolite) were found in both types of over-expressing lines (*35S:3ox2* and *LS1:3ox2*) compared to wild-type plants (Table 1). Transformants with a more elongated phenotype also exhibited the highest levels of GA_1_, thus evidencing a positive correlation between increased levels of bioactive GAs and internode growth. No apparent differences in GA_53_, GA_44_ and GA_19_ (GA_1_ precursors) were detected (Table 1).

**Table 1:** Levels of endogenous GAs in transgenic lines.

Line	GA_44_	GA_19_	GA_20_	GA_29_	GA_1_	GA_8_
wild-type	0.58	0.60	2.5	15.1	0.30	19.4
*35S:3ox2–10*	nd	0.58	0.13	3.2	0.63	27.1
*35S:3ox2–5*	0.56	0.73	0.1	nd	0.50	nd
*LS1:3ox2–18*	nd	nd	0.62	5.5	0.67	nd
*LS1:3ox2–3*	0.69	0.38	0.43	nd	0.40	11.7

Levels of endogenous GAs (ng·g^−1^ FW) in the shoot apices of the *35S:3ox2* and *LS1:3ox2* lines. GAs were quantified by GC-MS using internal standards.

nd = not determined.

None of the *StGA3ox2* over-expressing lines formed tubers under LD conditions but, when transferred to inducing SD conditions, they tuberized earlier than the controls (Figure 5D). The early tuberization response was significantly observed in both types of over-expressing lines (*35S:3ox2* and *LS1:3ox2*) (Table S1). Consistently, the yield in mass of tubers per plant was significantly higher in the over-expressing lines than in the controls (Table 2).

**Table 2:** Tuber yield of transgenic lines.

Line	n°tubers/plant	g of tubers/plant
wild-type	1.7±0.3	6.0±0.6
*35S:3ox2–10*	2.3±0.3	11.4±0.7 *
*35S:3ox2–5*	2.1±0.2	10.0±0.4 *
*LS1:3ox2–18*	1.5±0.3	7.5±0.3 **°**
*LS1:3ox2–3*	1.6±0.3	9.6±0.3 *
*LS1:3ox2–16*	2.5±0.4	3.2±0.2 *
*Tub1:3ox2–30*	3.6±0.3 *	5.6±0.4
*Tub1:3ox2–37*	3.2±0.4 *	6.5±0.3

Plants were grown for 4 weeks under SD inducing conditions before tubers were harvested. Data of number of tubers per plant and g of tubers are the average of 15 independent replicates for each transgenic line and 9 independent replicates for wild-type (± values indicate SE). Significant differences relative to wild-type are indicated with **°** (P<0.05) or with _*_ (P<0.01).

### Reduced levels of *StGA3ox2* in the aerial parts results in shorter plants and delayed tuberization under SD conditions

An exception in our analyses was the *LS1:3ox2*–16 line that was significantly shorter than the controls and in which reduced levels of *StGA3ox2* transcript were detected (Figure 5A–C), likely due to a co-suppression effect. Interestingly, the *LS1:3ox2*–16 plants tuberized significantly later than wild-type controls (Figure 5D; Table S1B). In addition, the co-suppressed *LS1:3ox2*–16 line produced smaller tubers and a lower tuber yield per plant (Table 2).

### Restricted over-expression of *StGA3ox2* in tubers slightly delays tuberization under SD conditions

**Figure 7:**
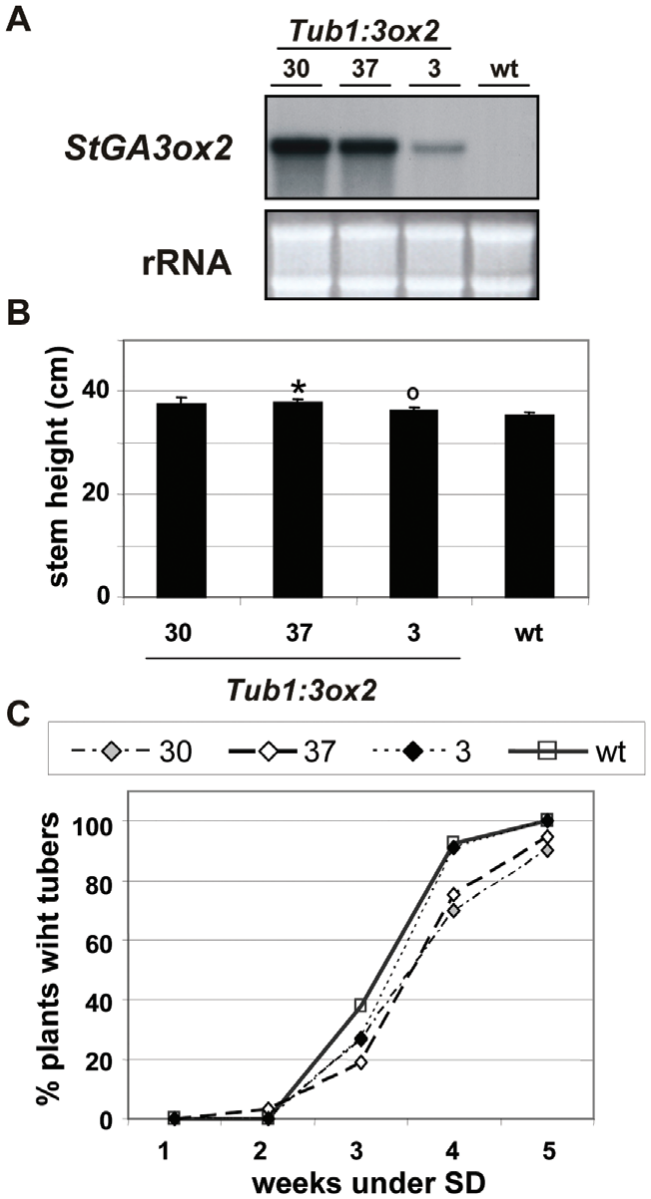
Over-expression of *StGA3ox2* under the tuber-specific (*Tub1:3ox2*) promoter. (**A**) RNA blot analysis from SD-grown tuber tissues, 30 µg of total RNA from each sample was electrophoresed and equal loading on the gel was assessed by ethidium bromide staining, a representative picture is shown out of at least two independent *Northern* analysis. (**B**) Stem heights of Tub1:3ox2 lines and wt (10 individual plants per line), measured after 3 weeks under SD tuber-inducing conditions. Errors bars indicate SE, asterisks (*) highly significant differences (P<0,01) and circles (**°**) significant differences (*t*-test; P<0,05) compared to wt. (**C**) Tuber induction data represent the average of five to nine independent replicates for each line from a representative experiment out of three with similar results.

The inverse correlation between the levels of *StGA3ox2* mRNA and the number of inductive SDs required for tuber formation found in *StGA3ox2* over-expressing and co-suppressed lines (Figure 5) is in contrast with the inhibitory role of GAs in regulating tuberization. To address whether high levels of *StGA3ox2* in the stolons inhibit tuberization, transgenic lines that over-expressed the *StGA3ox2* transcript only in tubers were obtained, by expressing its coding region under the patatin *B33* promoter control [29]. Several transformants (*Tub1:3ox2* lines) were regenerated that accumulated in tubers higher levels of the *StGA3ox2* mRNA than the controls (Figure 7A). Stem lengths of these lines were slightly longer or very similar to wild-type, with similar results obtained in measurements done in plants grown under LD conditions or after transferring them to SD to induce tuber formation (Figure 6 and 7B). Tuberization onset in the *Tub1:3ox2* over-expressers occurred at the same time or slightly delayed (but not earlier) compared to wild-type (Figure 7C; Table S1C). Tuber yield in these lines measured as mass of tubers per plant was similar to that of the controls. These plants, however, formed more tubers per plant compared to the controls (Table 2). Tubers were smaller and more elongated than those of control plants (data not shown), thus evidencing that the GA 3-oxidase activity does effectively over-accumulate in the transgenic tubers, with high levels of GA 3-oxidase activity affecting tuber growth and apical dominance by primarily formed tubers.

## Discussion

### The GA 3-oxidase activity plays a role in controlling both early and late responses after transfer to SD inductive conditions

We report the isolation of *StGA3ox2* and *StGA3ox1*, two potato cDNA clones encoding GA 3-oxidases (Figure 1A). The recombinant product of *StGA3ox2* is a functional enzyme catalyzing the conversion of both ^3^H-GA_20_ and ^14^C-GA_9_ into ^3^H-GA_1_ and ^14^C-GA_4_, respectively (Figure 3B). This agrees with pea *PsGA3ox1* and Arabidopsis *AtGA3ox2* genes encoding GA 3-oxidase proteins catalyzing the synthesis of active GAs both in the early- and non-C13-hydroxylation pathways [30], [31], [32]. The higher *StGA3ox2* expression in the shoots of the *35S:3ox2* and *LS1:3ox2* transgenic plants resulted in lower levels of GA_20_ (the substrate) and higher levels of GA_1_ (the product) than in control plants (Table 1), demonstrating that *StGA3ox2* encodes an enzyme with GA 3-oxidase activity also *in planta*. In addition, GA 3-oxidase activity might have a role in the control of bioactive GA synthesis during tuber induction, as we detected a strong down-regulated expression of *StGA3ox2* in stolons induced to tuberize, possibly contributing to the drop in GA_1_ levels observed in the stolons of plants exposed to SD tuber-inducing conditions (Figure 2B) [27].

One of the earlier events associated with the onset of tuberization is a change in the plane of cell division, from longitudinal to transversal, of the cells at the sub-apical region of the stolon. As a consequence of this switch, stolon growth is arrested and lateral tuber expansion begins [40], [41]. Polarized cell expansion in plant cells is controlled by cortical microtubules localized below the plasma membrane [42]. Orientation of these microtubules has been shown to be regulated by GAs, which promote a perpendicular distribution of the microtubules to the growing axis of the cell, thereby directing cell elongation along the longitudinal axis [43]. In induced stolons, decrease in GA_1_ levels partially caused by down-regulated expression of the *StGA3ox2* gene expression is thus likely to contribute to tuber formation, in concert with changes in the expression of other GA metabolism genes [27]. A complex regulatory mechanism at the transcriptional level has been proposed for such coordinated induction of *StGA2ox1, StGA20ox1, and StGA20ox3* expression and repression of *StGA3ox2* expression, after the switch from LD to SD conditions, possibly mediated by a common mechanism [27].

Transfer of plants to SD conditions induces at the same time a drop in the *StGA3ox2* mRNA levels in the stolons and a rise in the levels of transcript in the aerial part of the plant (shoot apex, leaves and the nodes; Figure 2B). These changes in gene expression are accompanied by an increase in stem elongation as a result of the reduced number of hours of light that the plants receive per day. While stem elongation is already observed after 1 week of transfer the plants to SD conditions, tuber formation requires at least 2 weeks under inductive conditions and, thereby, stem elongation was considered to be a short-term adaptive response independent of tuberization [3]. Our results suggest that up-regulated expression of *StGA3ox2* in the leaves might mediate this short-term elongation response. Alternatively, it might reflect the positive feed-back of a general reduction of bioactive GA levels in plants that have already stopped growing and started to senesce [3].

### Over-expression of *StGA3ox2* in the leaves results in elongated plants and early tuberization

We have investigated the function of a GA 3-oxidase in the regulation of tuber induction by generating transgenic potato plants that over-expressed *StGA3ox2* under the control of the constitutive *35S* CaMV (*35S:3ox2*) or the potato green tissue-specific *StLS1* (*LS1:3ox2*) promoters. As expected, transformants accumulating high levels of the transgene exhibited a taller phenotype due to longer internodes (Figure 5). Consistently, the co-suppressed line *LS1:3ox2–16*, with reduced *StGA3ox2* mRNA levels, showed reduced plant height with shorter internodes. Increase of plant height was similar in both constitutive (*35S:3ox2*) and leaf-specific (*LS1:3ox2*) over-expressers but always smaller than that observed in transformants over-expressing *StGA20ox1* [28]. This suggests that GA 20-oxidase activity may be limiting in potato plants (hence, the GA_20_ precursor) and/or that negative feed-back regulation of the genes encoding this enzyme is tighter than that of genes encoding GA 3-oxidase activity.

Over-expression of *StGA3ox2* induced early tuberization under SD inductive conditions, with a higher yield in mass of tubers per plant in the transgenic lines as compared to the wild-type controls (Table 2). Consistently, the co-suppressed line *LS1:3ox2–16* tuberized later and formed smaller tubers than the controls. Thus, a direct correlation between increased *StGA3ox2* expression in the leaves and early tuberization in SD was found in these lines, with those accumulating the highest levels of *StGA3ox2* mRNA (lines *35S:3ox2–10* or *LS1:3ox2–18*) also exhibiting the earliest tuberization onset. This observation is in apparent contradiction with the widely accepted notion that GAs inhibit tuberization. However, it is important to note that levels of GA_1_ in these transgenics were only determined in the shoots (Table 1) and we have been unable to quantify them in the stolons. To bypass this technical problem, we obtained transgenic lines that over-expressed *StGA3ox2* in tubers (*Tub1:3ox2* lines; Figure 7). These lines should accumulate higher levels of GA_1_ in tubers. Indeed, lines exhibiting increased levels of the *StGA3ox2* transcript in tubers showed a slight delay in the tuberization onset under SD conditions (Figure 7C). The patatin B33 promoter is a marker for tuber formation assumed to become active once the stolon starts to differentiate into a tuber [29]. Hence, the *Tub1:3ox2* lines should begin to accumulate GA_1_ in the stolons during their early transition into tubers, with this late increase in GA_1_ content having a mild impact on the tuberization onset. The *Tub1:3ox2* lines, however, formed a higher number of tubers per plant than the controls, but with reduced weight and with several small tubers of elongated shape attached to each stolon (Table 2). This phenotype indicates that the *StGA3ox2* product accumulated in the tubers of these lines, probably leads to increased GA_1_ content affecting tuber size and shape, as well as tuber apical dominance, with growth of secondary tubers observed in these lines. Although there are reports indicating that the patatin promoter is somehow leaky and some reporter expression can be observed in “pre-tuberizing” stolons [44], the subtle tuberization time phenotypes observed in *Tub1:3ox2* lines are likely to relate to the effect of altered GA levels on tuber development, whereas highly specific stolon promoters should be used to asses *StGA3ox2* photoperiodic control of tuber induction. Altogether, our results seem to suggest that increased levels of GA_1_ in stolons induced to tuberize have an inhibitory effect on tuber formation, in agreement with the accepted negative role of GAs in this process. Recent studies have suggested that GAs can move inside the plant, for instance a basipetal transport was observed in Populus and from leaves to the shoot apical meristem in Arabidopsis [45], [46], whereas upwards (acropetal) transport was described in pea stems [47].

Despite the subtle but significant phenotypes of our *StGA3ox2* over-expression lines, we performed grafting experiments between the wt and 35S:3ox2–5 transgenic line (data not shown). We could not detect any significant difference between the grafted specimens, possibly due to technical problems, such as earlier tuberization time in all grafts induced in older plants or by a tight regulation of GA_1_ metabolism and degradation possibly mediated by *StGA2ox1* [27].

### Contrasting effects on tuber induction time in *StGA3ox2* and *StGA20ox1* over-expressers: the importance of the different mobility of GA_20_ and GA_1_ in the plant and the balance between shoots and tubers in metabolizing GA_20_

Both *StGA20ox1* and *StGA3ox2* over-expressers display elongated stems and high levels of the bioactive GA_1_ in the shoots (Table 1; [28]). A relevant difference between these transgenic lines is the different accumulation in the shoots of GA_20_, the immediate precursor of the bioactive GA_1_: it was high in *StGA20ox1* over-expressers and it was low in *StGA3ox2* over-expressers. In addition, the early tuberization observed in the *35S:3ox2* and *LS1:3ox2* lines (Figure 5) is in clear contrast with previous results showing that over-expression of *StGA20ox1* results in delayed tuber formation under SD inductive conditions [28].

A possible explanation for this paradox is that GA_20_ and GA_1_ have different mobility within the potato plants, as occurs in pea. In this species, using *le* (blocked in GA 3-oxidase) and *na* (blocked prior conversion to GA_12_-aldehyde) mutants it was found that while GA_1_ application induces elongation of *le* lines, the stature of *le* is not altered when grafted to wild-type rootstock, indicating that GA_1_ is not transported in sufficient amount from the wild-type rootstock to the *le* scion. By contrast, stem elongation of the *na* line increased after GA application or by grafting to either wt or *le* rootstocks, indicating that GA_20_ or earlier precursors are readily transported from the wt or *le* rootstocks to the *na* scions, where they can be metabolized to GA_1_ [47]. In our transgenic *Tub1:3ox2* lines (Figure 7), *StGA3ox2* is over-expressed in tubers, thus likely accumulating higher local levels of GA_1_ (as deduced from the elongated tubers phenotype) and delaying tuberization; they however show slightly longer stem lengths or very similar to wt either under LD or after transferring them to SD conditions (Figs. 6, 7B), in agreement with less efficient acropetal GA_1_ transport.

In *35S:3ox2* and *LS1:3ox2* lines, over-expressing organs (aerial shoots) may act as a strong sink of GA_20_ in the plant transported acropetally from the stolons and roots, which results in increased GA_1_ levels and stem elongation; this sink effect lowers the level of this GA_1_ precursor in the induced stolons. As a consequence of this substrate decrease, the GA 3-oxidase activity in the stolons may produce less amount of the bioactive GA_1_ and tuberization occurs earlier. In *Tub1:3ox2* lines, by contrast, the absence of the GA_20_ sink effect by the aerial organs results in a local increase of GA_1_ delaying tuberization most likely by altering tuber development.

Together, our experiments provide evidence in support of different mobility for GA_20_ vs. GA_1_ that might contribute to tuberization time in potato. Furthermore, these conclusions would support that increased rates of stem elongation may occur concomitantly with an induction of tuber formation, as it was previously observed in *phyB* antisense plants, in which very elongated internodes (high levels of GA_1_ in shoots) and pale green leaves, co-existed with a strong induction of tuber formation [3], [48]. Conversely, reduced rates of stem elongation may occur concomitantly with an inhibition of tuberization, as it was observed in potato plants over-expressing Arabidopsis *CONSTANS* (*CO*) [4]. Both *PHYB* and CO are components of the photoperiod pathway that seem to interact with GA metabolism. However, since *StGA3ox2* over-expressing or co-suppressing lines still require SD photoperiods to produce tubers, this indicates that impairment of this transcript levels does not constitutively block and/or activate the photoperiod pathway, but modulates it.

In conclusion, we have shown that differential pattern of *StGA3ox2* expression in stolons plays a role in tuberization induction, unveiling a possible mechanism by which photoperiod- and GA-dependent pathways cross-talk in controlling potato tuberization. Our study also points out that the role of GAs in the regulation of photoperiod-controlled tuberization results from the combination of the enzymatic activities involved in their local production and metabolism, together with the differential distribution of GA_1_ and its precursors within the plant. It also highlights the regulatory role played by the integration of local levels of GAs with the photoperiod-dependent signal that induces tuberization.

## Supporting Information

Results S1 Isolation of potato GA 3-oxidase clones.
(PDF)

Figure S1 Negative feed-back regulation of the corresponding mRNA hybridizing to PCR product *B3ox*.
(PDF)

Figure S2 Structure of the *StGA3ox2* clones and amino acid sequence comparison to other GA 3-oxidases.
(PDF)

Table S1 Statistical analysis on the tuberization time in transgenic lines over-expressing *StGA3ox2*.
(PDF)
