# Supplementary material for: Gibberellin A1 Metabolism Contributes to the Control of Photoperiod-Mediated Tuberization in Potato
Source: PLoS One. 2011 Sep 22;6(9):e24458. doi: 10.1371/journal.pone.0024458 (PMC3178525; doi:10.1371/journal.pone.0024458)
Supplement: Results S1 — Isolation of potato GA 3-oxidase clones. (PDF) [file pone.0024458.s001.pdf]

Results S1

Results S1. Isolation of potato GA 3-oxidase clones

Using degenerated oligonucleotides (H5 and H7) a fragment of the expected molecular weight (460 bp), named *B3ox*, was amplified, cloned and sequenced, sharing 57% identity at the amino acid level with the protein At3ox2. The *B3ox* fragment exhibited some of the conserved regions present in all plant dioxygenases as well as certain motifs specific to the *GA3ox* genes (Figure S1). Furthermore, *B3ox* hybridized to a messenger exhibiting negative feed-back regulation by GA_3 (Figure S1), suggesting that this partial clone corresponds to a GA 3-oxidase-encoding transcript. Using the *B3ox* fragment as a probe, a cDNA library from leaves of the potato *ga1* mutant (Carrera *et al.*, 1999) was screened. We obtained several clones (Figure S2A) that were copies of the same gene, because they shared identical nucleotide sequences in their overlapping regions. In addition, two other full-length cDNAs were isolated which included an unspliced intron in the protein coding region. The presence of this intron resulted in a shift in the protein reading frame, allowing for a truncated protein that lacked any catalytic activity. Unspliced *StGA3ox2* RNAs seemed to be relatively abundant as several of the isolated clones contained such intron.
