# Supplementary material for: Gibberellin A1 Metabolism Contributes to the Control of Photoperiod-Mediated Tuberization in Potato
Source: PLoS One. 2011 Sep 22;6(9):e24458. doi: 10.1371/journal.pone.0024458 (PMC3178525; doi:10.1371/journal.pone.0024458)
Supplement: Figure S1 — Negative feed-back regulation of the corresponding mRNA hybridizing to PCR product B3ox. (PDF) [file pone.0024458.s002.pdf]

Figure S1

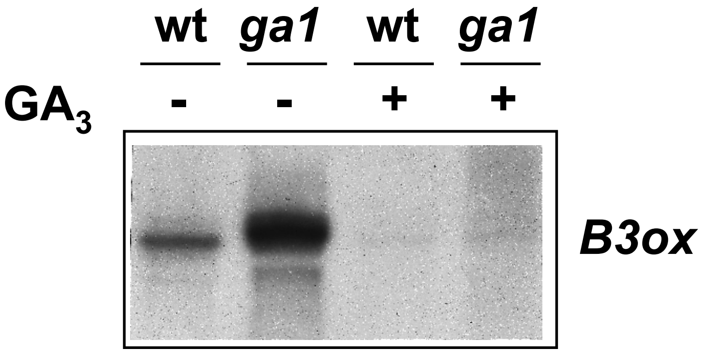

Figure S1. Negative feed-back regulation of the corresponding mRNA hybridizing to PCR product *B3ox*. Leaves and stems of 10-leaf plants were sprayed for 2 d with GA_3 or mock solutions. GA_3 was used at a final concentration of 50 μM. The *B3ox* (see Fig.2) corresponding transcript expression is increased in the *ga1* mutant. Reduction of its abundance by GA_3 application in both wild-type and *ga1* mutant plants confirms that the *B3ox* corresponding transcript is under negative feed-back regulation by GA_3, behaving similarly as others *GA20ox* and *GA3ox*.
