# Supplementary material for: Gibberellin A1 Metabolism Contributes to the Control of Photoperiod-Mediated Tuberization in Potato
Source: PLoS One. 2011 Sep 22;6(9):e24458. doi: 10.1371/journal.pone.0024458 (PMC3178525; doi:10.1371/journal.pone.0024458)
Supplement: Figure S2 — Structure of the StGA3ox2 clones and amino acid sequence comparison to other GA 3-oxidases. (PDF) [file pone.0024458.s003.pdf]

Figure S2

A

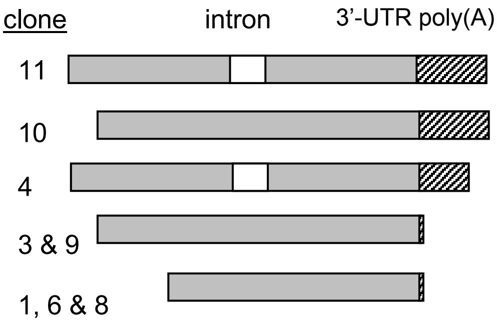

B

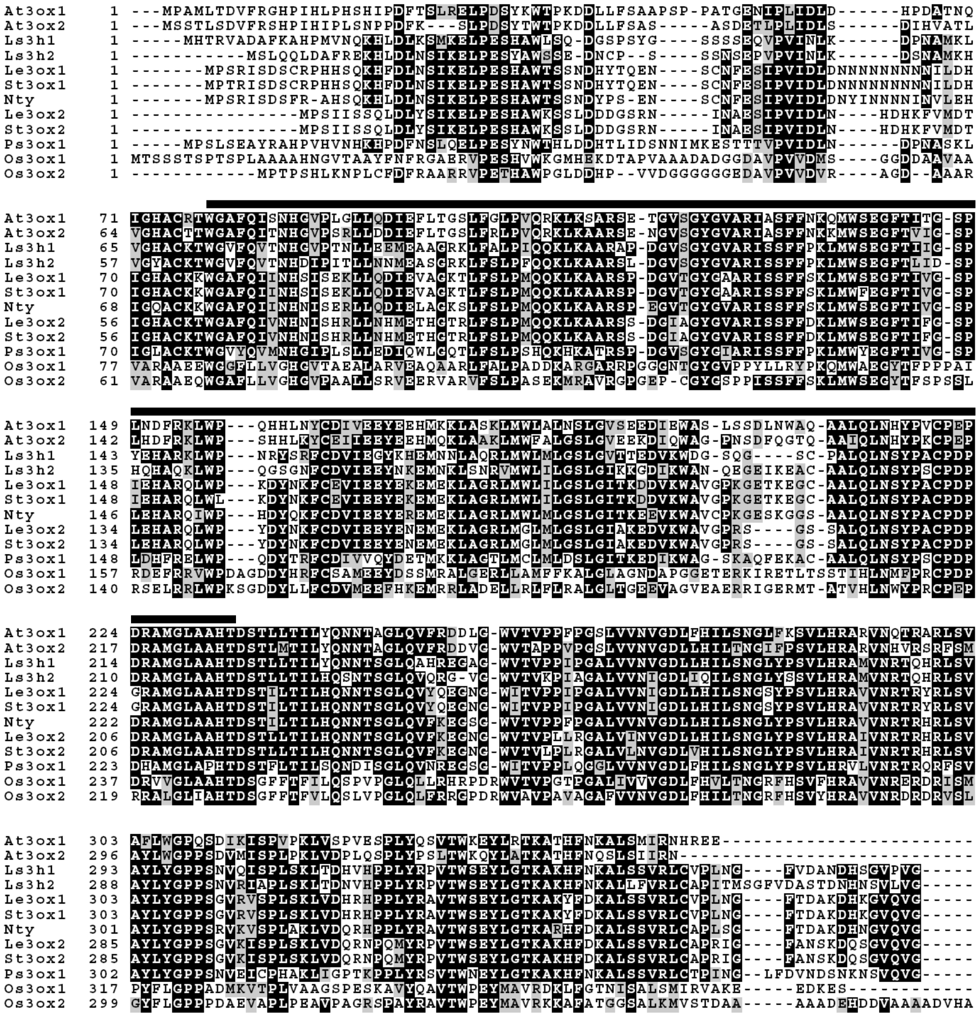

Figure S2. Structure of StGA3ox2 clones isolated from potato. **(A)** Schematic representation of the different clones corresponding to *StGA3ox2* mRNA. An unspliced intron is present in clones 11 and 4. Multiple poly-adenylation sites are used during transcription, with a poly-A tail added directly after the stop codon in clones 3 & 9, or clones 1, 6 & 8. **(B)** Comparison of potato *StGA3ox1* and *StGA3ox2* amino acid sequences with other GA 3-oxidases from Arabidopsis (*At3ox1* NM_101424 and *At3ox*2 NM_106683), pea *(Ps3ox1* AF010167), lettuce *(Ls3h1* AB012205 and *Ls3h*2 AB012206), tomato *(Le3ox1≈Sl3ox1* AB010991 and *Le3ox2≈Sl3ox2* AB010992), tobacco *(Nty* AB032198) and rice *(Os3ox1* AB054084 and *Os3ox2* AB056519) genes. . Black boxes indicate identical residues, and gray boxes indicate conserved residues. Dashes indicate gaps in the sequences. Sequence corresponding to the *B3ox* fragment is shown on top of the alignment with a thick black line.
