# Supplementary material for: Gibberellin A1 Metabolism Contributes to the Control of Photoperiod-Mediated Tuberization in Potato
Source: PLoS One. 2011 Sep 22;6(9):e24458. doi: 10.1371/journal.pone.0024458 (PMC3178525; doi:10.1371/journal.pone.0024458)
Supplement: Table S1 — Statistical analysis on the tuberization time in transgenic lines over-expressing StGA3ox2. (PDF) [file pone.0024458.s004.pdf]

Table S1

Statistical analysis on the tuberization time in transgenic lines over-expressing *StGA3ox2*.

A

Line \ Statistics	2-Way ANOVA*	Survival Curve Logrank test**
**Wt vs *35S:3ox2*-10**	**P<0.0001**	**P=0.11**
**Wt vs *35S:3ox2*-5**	**P<0.0001**	**P<0.05**

B

Line \ Statistics	2-Way ANOVA*	Survival Curve Logrank test **
**Wt *vs* *LS1:3ox2*-18**	**P=0.0001**	**P<0.05**
**Wt *vs* *LS1:3ox2*-16**	**P<0.0001**	**P<0.0001**

C

Line \ Statistics	2-Way ANOVA*	Survival Curve Logrank test**
**Wt *vs* *Tub1:3ox2*-30**	**P=0.50**	**P<0.01**
**Wt *vs* *Tub1:3ox2*-37**	**P<0.01**	**P=0.11**
**Wt *vs* *Tub1:3ox2*-3**	**P=0.20**	**P=0.052**

Table S1. Statistical analysis of tuberization time of wild type plants (Wt) vs different transgenic lines over-expressing *StGA3ox2*: constitutive *35S:3ox2* lines (**A**), leaf specific *LS1:3ox2* lines (**B**), and tuber specific *Tub1:3ox2* lines (**C**). *The ratio of individuals with tubers was weekly scored from three different experiments, and a two way ANOVA Pvalue for interaction between genotype and tuberization time is given. **The number of individuals with tubers vs time was plotted as a Survival Curve from one typical experiment, and the Logrank test generated a Pvalue testing the null hypothesis that the survival curves are identical. These statistical analysis were performed using the GraphPad Prism software package.
